# Long-lived states to sustain SABRE hyperpolarised magnetisation†
†Electronic supplementary information (ESI) available: Experimental details, sample specification, substrate characterisation, SABRE studies, pulse sequence details, simulations. See DOI: 10.1039/c6cp02844f



**DOI:** 10.1039/c6cp02844f

**Published:** 2016-08-15

**Authors:** Soumya S. Roy, Peter J. Rayner, Philip Norcott, Gary G. R. Green, Simon B. Duckett

**Affiliations:** a Department of Chemistry , University of York , Heslington , York , YO10 5DD , UK . Email: simon.duckett@york.ac.uk ; Tel: +44 (0)1904 322564; b York Neuroimaging Centre , The Biocentre , York Science Park , Innovation Way , Heslington , York , YO10 5DD , UK

## Abstract

The applicability of the magnetic resonance (MR) technique in the liquid phase is limited by poor sensitivity and short nuclear spin coherence times which are insufficient for many potential applications. Here we illustrate how it is possible to address both of these issues simultaneously by harnessing long-lived hyperpolarised spin states that are formed by adapting the Signal Amplification by Reversible Exchange (SABRE) technique. We achieve more than 4% net ^1^H-polarisation in a long-lived form that remains detectable for over ninety seconds by reference to proton pairs in the biologically important molecule nicotinamide and a pyrazine derivative whose *in vivo* imaging will offer a new route to probe disease in the future.

## Abstract 2

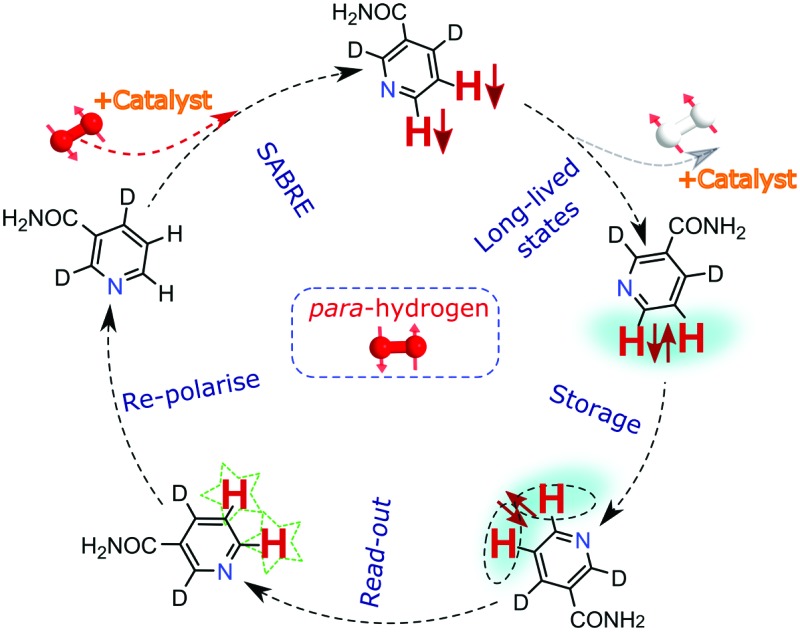
More than 4% net ^1^H-polarisation is created, in seconds, that is detectable for over 2 minutes.

## Introduction

Over the last few decades, Nuclear Magnetic Resonance (NMR) and Magnetic Resonance Imaging (MRI) have evolved as two extremely important techniques that have applications in almost all branches of science, ranging from molecular studies to human imaging.^1–3^ Even after this tremendous success, the applicability of these techniques is limited by sensitivity in general and relaxation in some cases.^4^ Low sensitivity is derived from the fact that nuclei possess little intrinsic magnetisation and interact weakly with a magnetic field. Measurement perturbs the initial Zeeman alignment, which then relaxes to re-establish the original state, for signal averaging purposes in a process whose major contribution is derived from dipolar interactions for nuclei like protons.^5^ Several techniques, known by the term hyperpolarisation, are being developed to tackle this sensitivity issue, with molecular symmetry and deuteration often being harnessed to reduce the rates of relaxation.^6,7^

In this report we use the Signal Amplification by Reversible Exchange (SABRE) process^8^ to produce >4% net ^1^H-polarisation and then transfer it into a longer-lived coherence. The SABRE hyperpolarisation technique operates over seconds and is therefore fast in creating its hyperpolarisation when compared to the methods of Dynamic Nuclear Polarisation (DNP)^9^ and optical pumping.^10^ It harnesses *para*-hydrogen (*p*-H_2_) as the latent source of polarisation and unlike the traditional hydrogenative method of Weitekamp,^11^ Eisenberg,^12^ and Bargon^13^ it does not rely in changing the chemical identity of the hyperpolarised probe. This method is simple to perform and has been successfully automated to ensure reproducibility.^14^ Since its inception in 2009,^8^ it has been shown to successfully hyperpolarise various nuclei^15–20^ including ^1^H, ^13^C, ^15^N, ^19^F, ^31^P. Among these, ^1^H holds special attention as almost all imaging applications^3^ and methodologies are based on it for the reasons of sensitivity and accessibility. Predicted refinements^21^ on the transfer process have included radio frequency (r.f.) driven transfer at low^17,22^ and high field^23^ with spontaneous transfer being reported between 0 G and 200 G.^8^

The lifetime of the nuclear singlet associated with *p*-H_2_ is in excess of 1 year in the absence of a quenching agent.^24^ This molecule reflects an example of how *T*_1_ should not be thought of as limiting factor in magnetic state lifetime. In 2004, Levitt and co-workers harnessed this property in a related molecule containing a ^1^H pair to show that it was possible to create a similar pseudo-singlet state through the application of an r.f. pulse sequence and to detect them several minutes later.^25,26^ This breakthrough has stimulated significant interest in harnessing such states, known as long-lived singlet states (LLS), more widely because of their potential as clinical imaging probes.^27^ These probes harness the fact that there are specially correlated quantum states within coupled nuclei that can have longer lifetimes than their individual *T*_1_ values.^28^ In this context, a true singlet state is anti-symmetric with respect to particle interchange, and as in the case of *p*-H_2_ does not couple further outside the two spin system whilst being immune to dipolar relaxation.^28^ A large range of pseudo-singlet states have now been created that do not meet all of these requirements, although they can have long-lifetimes.^29,30^ There are also reported examples of true singlets that are created in a chemically equivalent but magnetically inequivalent spin-pair.^31,32^ We use the term singlet to refer to both here.

**Fig. 1:**
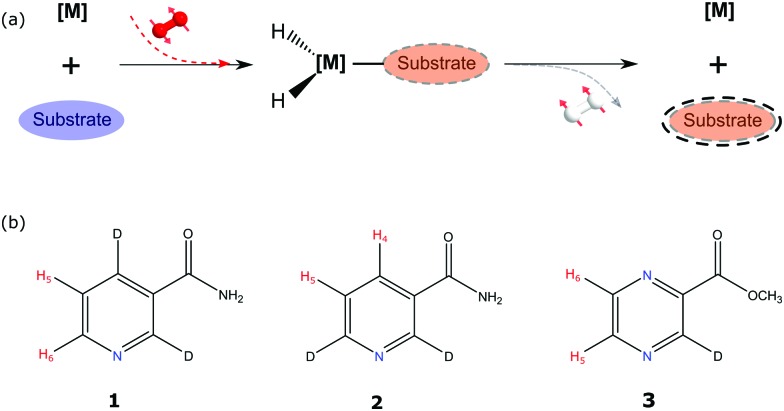
Schematic representation of: (a) the SABRE process and (b) the structures of the substrates 2,4-*d*_2_-nicotinamide (**1**), 2,6-*d*_2_-nicotinamide (**2**) and methyl-3-*d*-pyrazine-2-carboxylate (**3**) studied in this report with the protons labelled appropriately.

**Fig. 2:**
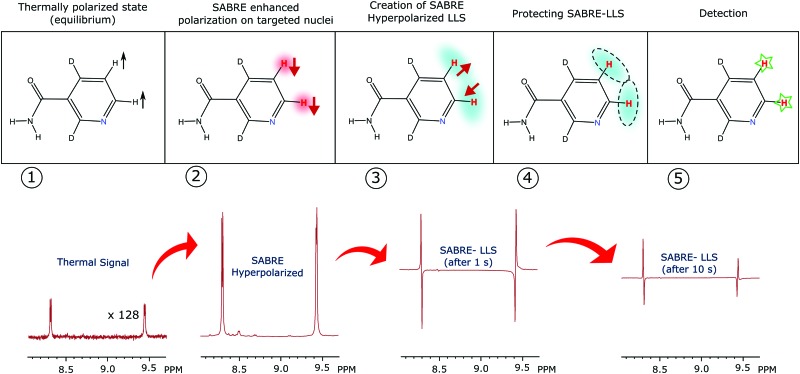
SABRE-LLS scheme depicting the different stages of the process (upper conceptual): (1) signal resulting for the targeted substrate protons under thermal equilibrium, multiplied by 128. (2) Hyperpolarised magnetisation detected with a single scan after SABRE. (3) Hyperpolarised magnetisation is converted into singlet order by applying the r.f. pulse sequence shown in Fig. 5 and then converting it back into observable magnetisation after a 1 s spin-lock. (4) The created singlet state is protected *via* a spin-lock for a period ranging from seconds to minutes. (5) Readout after 10 s of spin-lock.

Fig. 1 illustrates the process of SABRE in conceptual form. A metal complex acts to bind *p*-H_2_ and the hyperpolarisation target. The latent magnetism of *p*-H_2_ can then be productively harnessed if the two hydride ligands become magnetically distinct whilst retaining their original spin order in this transient product. Over the next few seconds, evolution under the coupling Hamiltonian leads to transfer of this spin order into the product which can be retained after its dissociation from the metal complex. In this report we use this approach to create highly polarized longitudinal magnetisation in the three heterocyclic molecules that are shown in Fig. 1. These molecules exhibit low-toxicity^33^ and play a role in biological processes such as NADH synthesis.^34^ Furthermore, their derivatives also feature in various antibiotics.^35–37^ Partial deuteration of these substrates is used to produce the desired pair of coupled spin-1/2 nuclei for this study. Immediately after creating highly polarized magnetisation in these substrates under SABRE, we convert it into a singlet state by applying a series of r.f. pulses in a manner that is optimised for the individual spin-system according to the method of Levitt.^26^ As this singlet represents an unusual non-magnetic form, its detection is optimised through a specific r.f. driven readout step. The complete experimental scheme of SABRE-LLS is shown pictorially in Fig. 2. We also employ a refocussing step to convert the antiphase signal of Fig. 2 into more useful in phase polarisation.

## Theoretical background

### SABRE

The method used here to create the initial hyperpolarised state is based on the SABRE technique for which a firm theoretical basis exists.^38,39^ While it is very challenging to emulate the whole system theoretically, subtle approximations such as the level anti-crossing (LAC) approach^40–42^ lead to a more intuitive perspective. In this report we continue with density matrix based numerical approach to describe and quantify the different types of magnetisation that are created. For simplicity, we consider the two ^1^H nuclei in a single substrate molecule to couple to two *para*-hydrogen derived hydride nuclei on the metal centre. This results in an active 4-spin system. In isotropic liquid state, the Hamiltonian of such a spin-1/2 system, in a magnetic field, can be written as:1
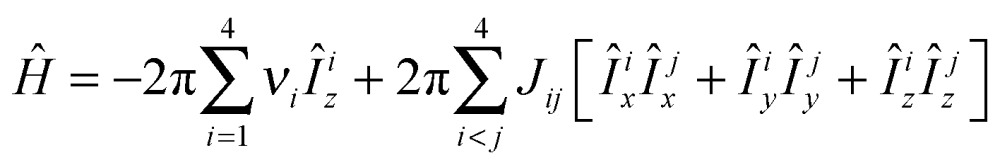
where *ν*_*i*_ is the Larmor frequency of the *i*-th spin and *J*_*ij*_ is the scalar coupling constant between spin-*i* and spin-*j* in Hertz. *Î* *i* *z* and *Î* *j* *z* denote the *i*-th spin and *j*-th spin angular momentum operators in the *z*-direction. Initially, the hydride ligands possess singlet spin order which can be written as the Cartesian product operator:2
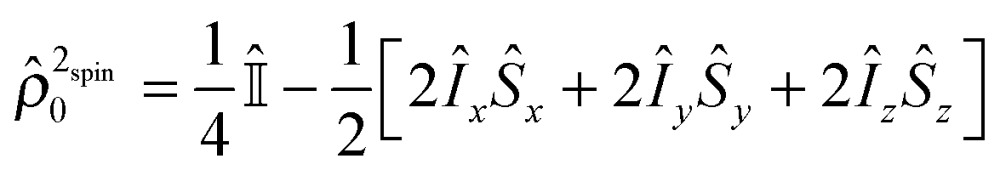
where, *I* and *S* denote the two spin angular momentum operators and 
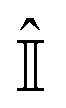
 is the unity operator. The spins of the substrate are denoted by R and T and their labels were chosen without implying spin-topological resemblance.

**Fig. 3:**
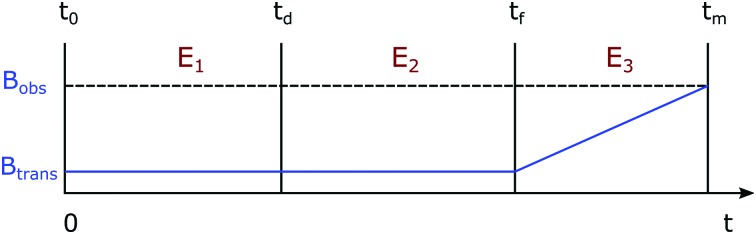
The variation of magnetic field in the SABRE experiment as a function of time. *E*_1_, *E*_2_, and *E*_3_ denote three evolution periods during the time intervals (*t*_0_ – *t*_d_), (*t*_d_ – *t*_f_), and (*t*_f_ – *t*_m_) respectively. *B*_trans_ and *B*_obs_ represent transfer and observation magnetic field strengths respectively.

The SABRE process can be divided into three stages: (i) evolution of the resulting 4-spin system in the inorganic template at a defined transfer field, (ii) evolution of the two substrate spins after its dissociation from the template in the transfer field, and (iii) evolution of substrate spins during dynamic field transfer into the spectrometer where their r.f. encoding is achieved. A schematic diagram of this process is shown in Fig. 3.

The initial two-spin singlet order of *p*-H_2_ changes into that of a coupled four spin system as soon as the template forms. In this model, any contribution from the thermally polarised spins of the substrate is neglected, such that the initial state for subsequent evolution can be written as:3
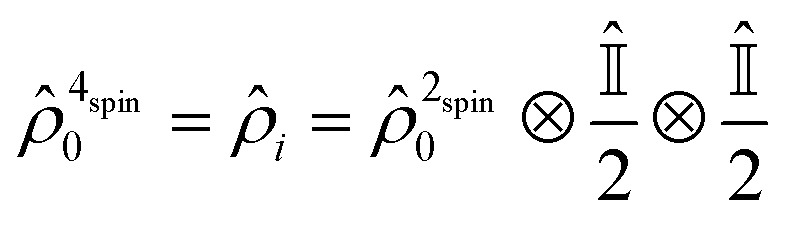
Here 
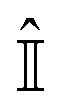
 denotes a 2 × 2 identity matrix. The time evolution of *ρ*_*i*_ is then determined by solving the Liouville–von Neumann equation. The solution can be written as:

4 *ρ̂*(*t*) = exp(–*iĤt*)*ρ̂*_*i*_ exp(+*iĤt*)

This evolution is considered to take place during the time the four spins reside on the template, defined by the dissociation time, *t*_d_, in a specified transfer field (*B*_trans_). The resulting density matrix can then be represented in the more intuitive product-operator formalism^43^ as,5
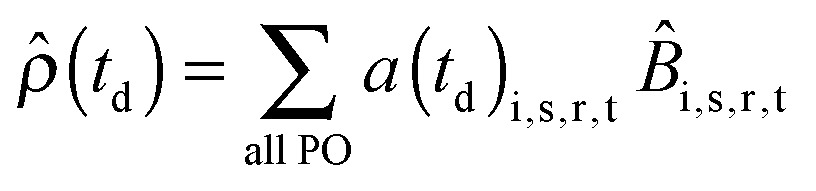
where *a*(*t*_d_)_i,s,r,t_ are the time dependent amplitudes of the product operators *B*_i,s,r,t_ of the 4-spin system. The subscripts i, s, r and t are the spin-labels of these spins and all PO means a summation over all product operators. The resulting amplitudes at time *t*_d_ can be determined as,

6 *a*(*t*_d_)_i,s,r,t_ = Tr[*ρ̂*(*t*_d_)**·***B*_i,s,r,t_]

At this point dissociation of the substrate from the metal centre takes place and the free substrate now reflects an isolated two-spin system which is still evolving in the transfer field. Neglecting relaxation, the density matrix after a time *t*_d_ can now be written as the sum of the remaining coherence orders,

7 *ρ̂*(*t*_d_|*t*_2_) = *a*_*R*_*z*_*T*_*z*__2*R*_*z*_*T*_*z*_ + *a*(*t*_2_)ZQ_*x*_ + *b*(*t*_2_)ZQ_*y*_ + *c*(*t*_2_)*R*_*z*_ + *d*(*t*_2_)*T*_*z*_

where zero-quantum terms are defined as; ZQ_*x*_ = 2*R*_*x*_ *T*_*x*_ + 2*R*_*y*_ *T*_*y*_ and ZQ_*y*_ = 2*R*_*y*_ *T*_*x*_ – 2*R*_*x*_ *T*_*y*_. The coefficients of these terms can be calculated by solving a series of coupled differential equations as detailed by Green *et al.* ^39^

In the third step, the substrates spins evolve further under transfer through the dynamic field that takes them into the observation field (*B*_obs_). The Hamiltonian and density matrix at this point are represented in the interaction picture:

8 *ĤI*1(*t*) = exp(+*iĤ*_0_*t*)*Ĥ*_1_(*t*)exp**(–***iĤ*_0_*t*)

9 *ρ̂*^*I*^(*t*_d_,*t*_f_|*t*) = exp(+*iĤ*_0_*t*)*ρ̂*(*t*_d_,*t*_f_)exp(–*iĤ*_0_*t***)**

where *Ĥ*_0_ is the initial Hamiltonian. Following the same procedure as described above, it can be shown that at the point of r.f. excitation, *t*_m_, they now have the form:^38^

10 *ρ̂*(*t*_d_,*t*_f_|*t*_m_) = *a*_*R*_*z*_*T*_*z*__2*R*_*z*_*T*_*z*_ + *a*_m_(*t*_f_|*t*_m_)ZQ_*x*_ + *b*_m_(*t*_f_|*t*_m_)ZQ_*y*_ + *c*(*t*_f_)*R*_*z*_ + *d*(*t*_f_)*T*_*z*_

During this synchronous process, both *a*_m_(*t*_f_|*t*_m_) and *b*_m_(*t*_f_|*t*_m_) average to zero such that the final state becomes:

11 *ρ̂*_m_(*t*_f_) = *a*_*R*_*z*_*T*_*z*__2*R*_*z*_*T*_*z*_ + *c*(*t*_f_)*R*_*z*_ + *d*(*t*_f_)*T*_*z*_

**Fig. 4:**
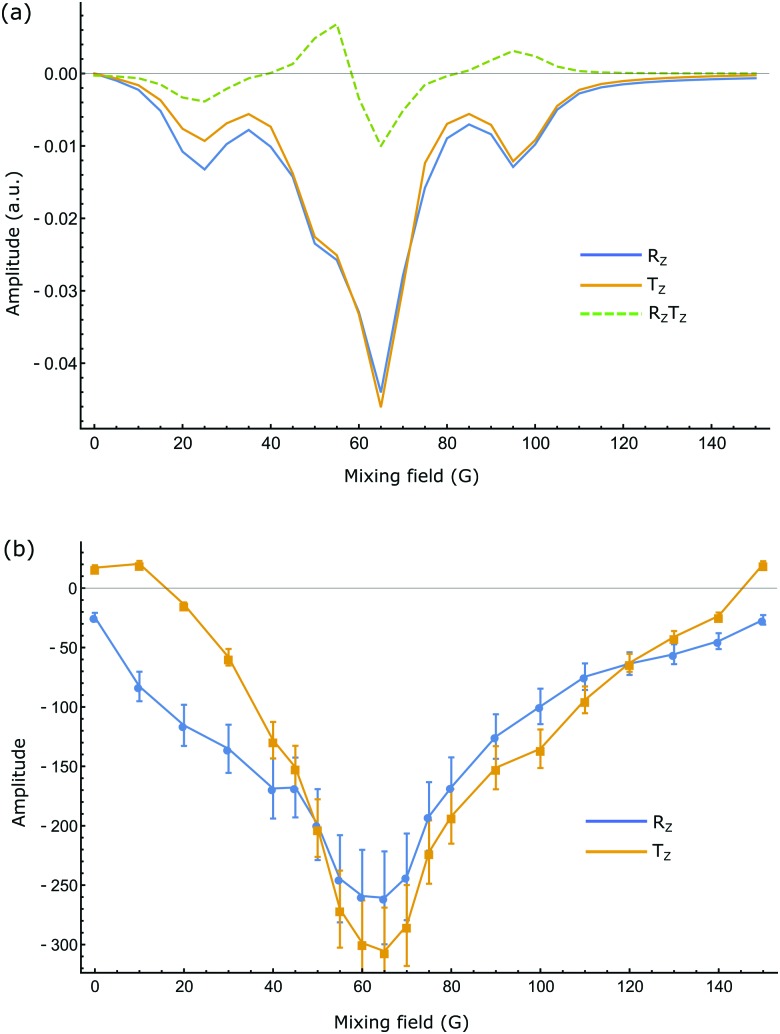
(a) Simulated and (b) experimental values of SABRE amplitudes of **1** as a function of mixing field. Blue and orange represent single quantum longitudinal magnetisation (*R*_*z*_ and *T*_*z*_) of proton pair in the system. Dotted curve in (a) depicts two-spin order term (*R*_*z*_ *T*_*z*_). Experimental points are shown with their respective error bars.

The numerical evaluations of the substrates indicated in Fig. 3 were completed using appropriate routines in Mathematica^44^ and a typical set of results for compound **1**, are shown in Fig. 4. These calculations show that the SABRE process generates significant populations of single spin longitudinal magnetisation and minor populations of the corresponding two-spin order term. Despite the simplified treatment associated with a 4-spin system, good agreement with experiment is seen.

### Long-lived singlet states (LLS)

**Fig. 5:**
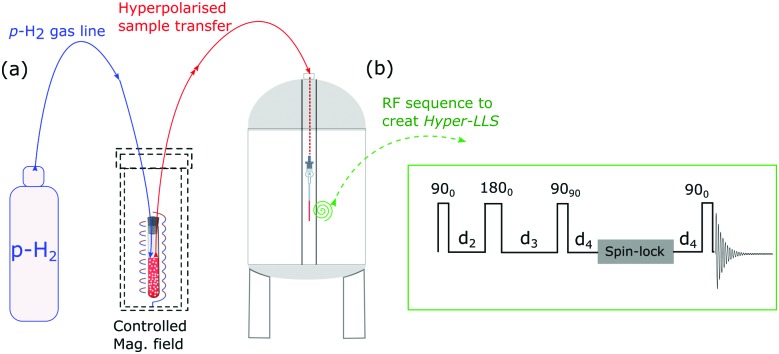
(a) Schematic illustration of the experimental procedure associated with the SABRE hyperpolarisation technique; (b) pulse sequence used to create, store and read-out singlet states with 
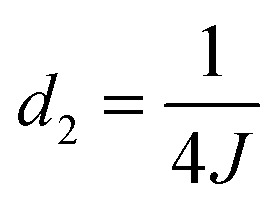
, 
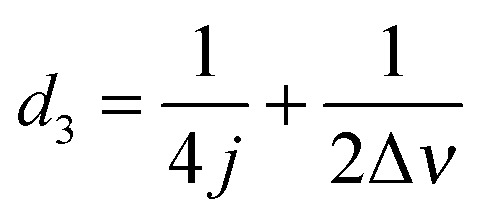
 and 
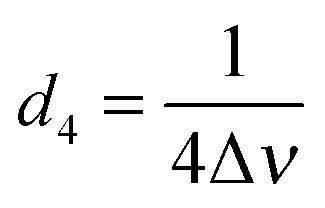
, where *J* and Δ*ν* denote scaler-coupling constant and chemical shift difference between two spins in Hertz respectively. We used a Waltz-16 composite pulse of 1 kHz amplitude as the spin-lock. Parameter values set according to Table 2.

The r.f. pulse sequence used in this study to create a LLS, and its subsequent detection, is shown in Fig. 5. The first part of the LLS pulse sequence converts the enhanced amplitude of longitudinal magnetisation into a mixture of singlet (|S) and triplet (|T_0_) states, as defined below in terms of Cartesian product operator formalism:12
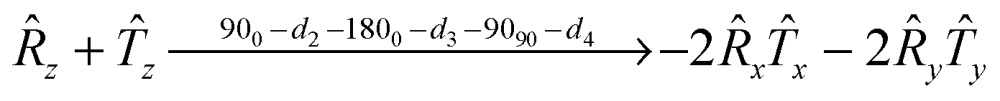
The parameters in the pulse sequence are defined in Fig. 5b. The resulted singlet–triplet mixture is then subject to a low-powered ‘spin-lock’, during which the triplet terms quickly equilibrate whereas the singlet does not interconvert with the triplets as this process is symmetry forbidden. As such singlets are non-magnetic, a read-out step is required to extract observable magnetisation. Neglecting relaxation, the later part of the pulse sequence leads to,13
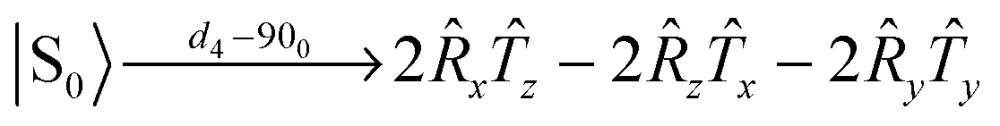
The anti-phase terms produce a typical ‘up-down-down-up’ pattern spectra as reported in the experimental section which can be refocussed to produce in-phase signal.

## Experimental section

A schematic picture of the experimental procedure used here is shown in Fig. 5. All the associated experiments were performed on a 400 MHz Bruker Avance III series spectrometer at 298 K. Three substrates, 2,4-*d*_2_-nicotinamide (**1**), 2,6-*d*_2_-nicotinamide (**2**) and methyl-3-*d*-pyrazine-2-carboxylate (**3**) were used in this study. They were polarised under SABRE *via* the precatalyst [IrCl(COD)(IMes)] which was employed at a 5 mM concentration in methanol-*d*_4_. The substrate loadings were varied from 5–40 equivalents based on iridium. These samples were then examined in a 5 mm NMR tube, under 3 bars of *p*-H_2_ or in an automated polariser that has been described previously.^45^ The automated polariser achieves magnetisation transfer in a predefined magnetic field which can be selected to lie between 0 and 150 G. Other details of the experimental scheme, sample specification and characterisation can be found in the ESI.†

## Results

A study of the field dependence exhibited by each substrate on the degree of SABRE enhancement was first undertaken. For substrate **1**, an average maximum 285-fold enhancement was observed for its two spins in a mixing field of 65 G when there were 5 equivalents of ligand relative to iridium in the flow system described in the experimental. This sample loading produced an enhancement of 1090 fold when measured *via* ‘shake & drop’ technique due to better H_2_ transport. Table 1 summarizes the enhancement results for all three substrates, at two different sample concentrations (see later for the results of a systematic study of varying the sample loads from 5 to 40 equivalents relative to the catalyst, while keeping all the other parameters unchanged). The close match between the simulated and experimental results (Fig. 4) confirms the generation of large single-spin amplitudes through SABRE.

**Table 1:** Signal enhancement and relaxation data associated with **1**, **2** and **3** under SABRE in a 5 mm NMR tube with the indicated catalyst: substrate ratios

Substrate	**1**		**2**		**3**	
Catalyst: substrate ratio	1 : 5	1 : 20	1 : 5	1 : 20	1 : 5	1 : 20
Enhancement factor (by shake & drop)	H5: –1025 ± 70	H5: –280 ± 50	H4: –860 ± 120	H4: –220 ± 50	H5: –1540 ± 220	H5: –460 ± 80
	H6: –1150 ± 80	H6: –300 ± 50	H5: +450 ± 110	H5: +90 ± 25	H6: –1230 ± 170	H6: –310 ± 65
*T*_1_ (s)	H5: 11.9 ± 0.4	H5: 11.4 ± 0.4	H4: 11 ± 0.5	H4: 10 ± 0.4	H5: 21 ± 1.7	H5: 20.2 ± 1.5
	H6: 7.6 ± 0.2	H6: 7.1 ± 0.3	H5: 11 ± 0.5	H5: 10.3 ± 0.4	H6: 17.9 ± 0.9	H6: 17.1 ± 0.5
*T*_LLS_ (s)	37 ± 4	38 ± 6	47 ± 6	49 ± 8	49 ± 7	45 ± 8

This polarisation was then converted into the corresponding singlet state by applying the first part of r.f. pulse sequence of Fig. 5. The resulting singlet polarisation was then stored over a spin-lock time that was varied from seconds to minutes. After this point, the later part of the pulse sequence was used to convert this polarisation into an observable form.

**Fig. 6:**
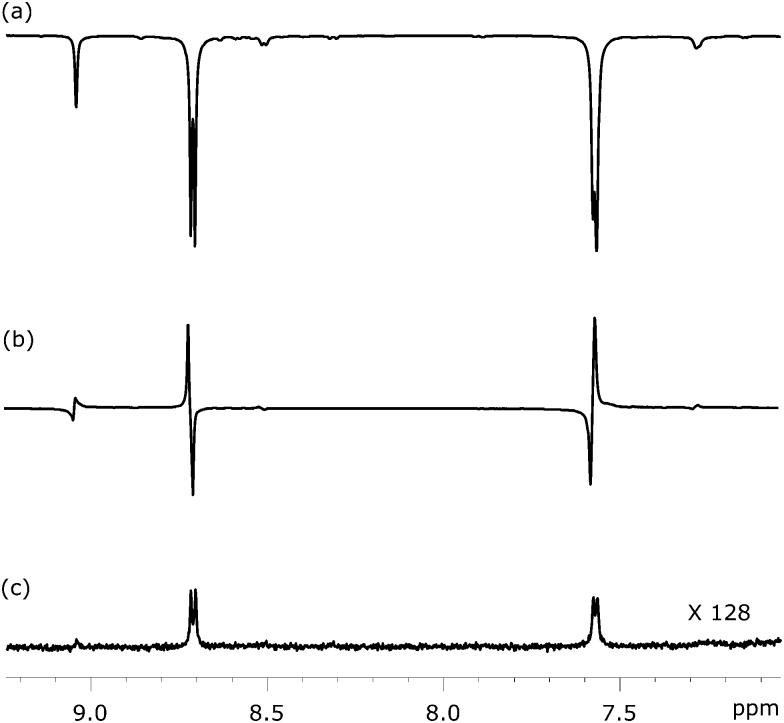
^1^H NMR spectra showing selected coherences associated with the pair of ^1^H spins in **1** at different stages of the process: (a) SABRE polarisation as achieved at a mixing field of 65 G, (b) LLS form created *via* SABRE driven longitudinal magnetisation using the pulse sequence shown in Fig. 5, and (c) ^1^H NMR spectra under thermal equilibrium for comparison purpose.

Fig. 6 shows three NMR spectra of substrate **1** that were recorded to illustrate this approach. Fig. 6a illustrates the SABRE enhanced polarisation that is ultimately used to create the LLS state. Fig. 6b then shows the resulting singlet readout, on the same vertical scale, after a 1 s spin-lock. The high efficiency of the singlet state creation and subsequent readout is therefore illustrated. For comparison purposes, Fig. 6c shows the corresponding thermally acquired spectrum with a 128 fold vertical expansion. Key experimental parameters that relate to application of this pulse sequence, for each substrate, are listed in Table 2.

**Table 2:** Details of spin system and associated experimental parameters for the substrates **1**, **2** and **3**

Substrate	**1**	**2**	**3**
*J*-coupling constant (Hz)	4.90	8.05	2.50
Chemical shift difference (Hz)	457.0	298.0	34.4
*d*_2_ (ms)	51.02	31.05	100.0
*d*_3_ (ms)	52.11	32.73	114.53
*d*_4_ (ms)	0.547	0.839	7.26

**Fig. 7:**
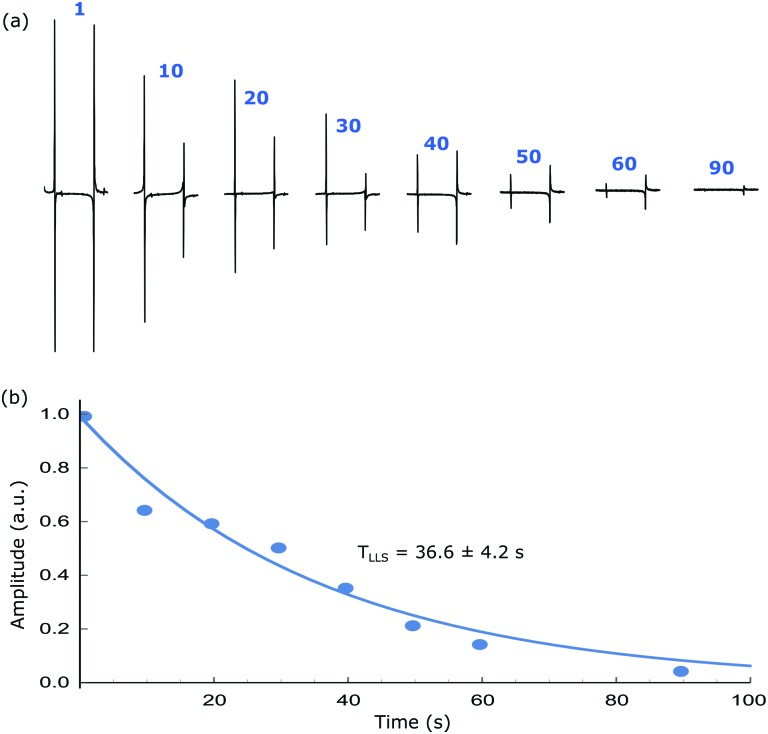
(a) Hyperpolarised singlet order based NMR spectra after the indicated spin-lock duration and (b) corresponding integration values showing the decay of signal due to relaxation. An exponential fit gives a *T*_LLS_ of 36.6 ± 4.2 s while *T*_1_ is around 10 s for the same system (**1**). All experiments are performed at a mixing field of 65 G and bubbling *p*-H_2_ for 10 s before transferring the sample into the high field for r.f. pulsing.

Singlet lifetimes (*T*_LLS_) were then measured by tracking the exponential decay of the read-out over an evolving spin-lock duration. The *T*_LLS_ values for the different samples are tabulated in Table 1, alongside the corresponding *T*_1_ values of their longitudinal magnetisation as measured by inversion-recovery. In all cases, the *T*_LLS_ lifetime exceeds that of *T*_1_ with the maximum values reaching 50 s for **2**. A typical *T*_LLS_ decay trace as a function of spin-lock duration is shown in Fig. 7 with the anti-phase magnetisation being readily visible at 90 s. When substrate **1** is examined it gives an S/N ratio of 2.5 at 90 s. In contrast when substrate **2** is examined the same S/N ratio is achieved after 120 s, while for **3** this point is achieved at 132 s. These data confirm that small chemical shift difference found between the protons in **3** is beneficial for the LLS lifetime even though the *J*_HH_ coupling that connects them is smaller than those of **1** and **2**.

**Fig. 8:**
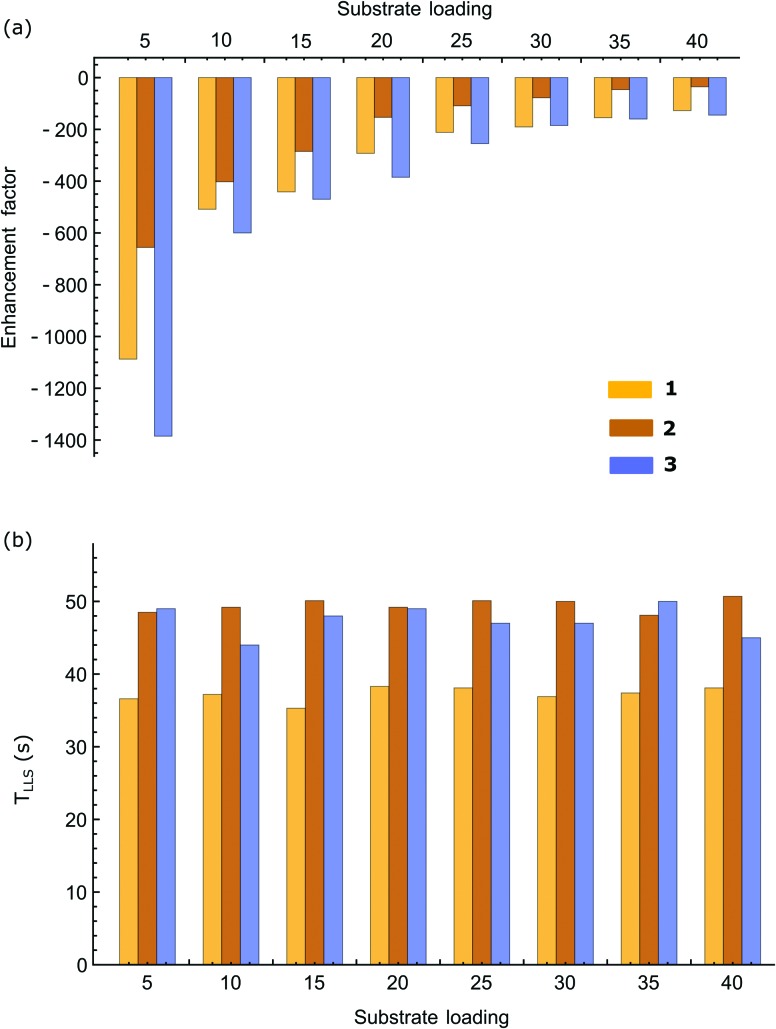
(a) Average enhancement factors for the two protons contributing to the singlet state, and (b) the singlet state lifetimes (*T*_LLS_) of substrates **1**, **2** and **3** as a function of their molar ratio to catalyst.

A systematic study of *T*_LLS_ was then carried out for all three samples over a range of sample loadings and the associated results are plotted in Fig. 8. No significant variation in *T*_LLS_ with substrate equivalent was observed in contrast to the enhancement factors which fall with greater substrate excess. This change is seen for all three substrates in accordance with the fact that *p*-H_2_ becomes a limiting reagent and the enhancement levels fall with higher loadings (greater spin dilution).

**Fig. 9:**
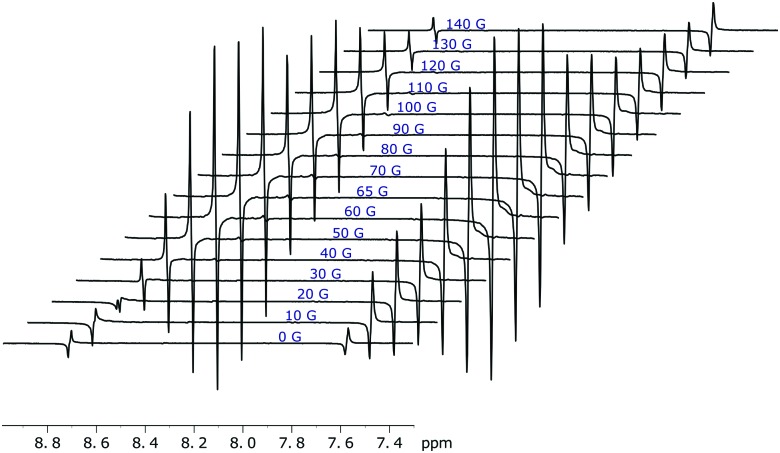
Hyperpolarised singlet order of **1** (with 5 equivalents of ligand) as generated over a range of magnetic mixing fields. Traces recorded using the pulse sequence shown in Fig. 5 with a spin-lock of 1 s. The highest singlet order amplitude is achieved at a 65 G mixing field.

The amount of singlet order created using this approach also depends on mixing field that generates the initial SABRE enhancement as detailed in Fig. 9. Not surprisingly, the maximum level of singlet polarisation is achieved at 65 G for **1** in agreement with the optimal single spin term generation by SABRE at this field.

The results of Fig. 8 and 9 confirm that achieving optimal hyperpolarisation, and a long *T*_LLS_ lifetime, requires the careful balancing of substrate loading and SABRE transfer field.

## Conclusions

*p*-H_2_ has already been successfully employed in generating nuclear spin hyperpolarisation in a range of different nuclei and species with the SABRE process allowing the hyperpolarisation target to be unchanged. Currently, these highly polarised nuclear spins are somewhat under-exploited due to their relatively low spin-state lifetimes which are often below 10 s for ^1^H and thus challenge the idea of *in vivo* applications. In this report we show a method to store highly polarised magnetisation in a specially created coherence order namely a singlet state which is immune to dipole–dipole relaxation and hence often has much longer lifetimes than the more usual *T*_1_. This feature makes singlet states particularly attractive for transporting nuclear hyperpolarisation in NMR and MRI applications and for reducing unwanted signal losses in experiments caused by undesirable fast nuclear relaxation.

We illustrate the creation of these states in a highly polarised form in variants of nicotinamide and pyrazine to demonstrate that singlet state polarisation can be unlocked through SABRE. In these molecules, the corresponding *T*_LLS_ values reach 50 seconds with a >1000 fold enhancement factor compared to the situation where the corresponding state is formed *via* a thermally polarised signal. These molecular prototypes exemplify the potential of SABRE to deliver highly polarized magnetisation with long lifetimes that may aid in future *in vivo* study. Recent work by Theis *et al.* has illustrated that similar long-lived ^15^N derived singlet states can be produced *via* SABRE with significant amplitudes.^46^ These two complementary studies therefore illustrate a simple route to hyperpolarised long lived magnetic states that we are now seeking to develop further for ^13^C-pairs. The enhancement factors and lifetimes presented here are clearly limited by the molecular architecture of these probes. We are seeking to improve on these agents, and the levels of ^1^H-hyperpolarisation that can be achieved, through further catalyst and substrate optimisation.
