# Supplementary material for: Long-lived states to sustain SABRE hyperpolarised magnetisation
Source: Phys Chem Chem Phys. 2016 Aug 15;18(36):24905–11. doi: 10.1039/c6cp02844f (PMC5436088; doi:10.1039/c6cp02844f)
Supplement: Supplementary file 1 [file CP-018-C6CP02844F-s001.pdf]

Electronic Supplementary Material (ESI) for Physical Chemistry Chemical Physics.
This journal is © the Owner Societies 2016

# Long-lived States to Sustain SABRE Hyperpolarised Magnetisation

Soumya S. Roy,[a] Peter J. Rayner,[a] Philip Norcott,[a] Gary G.R. Green[b] and Simon B. Duckett[a]

[a] *Department of Chemistry, University of York, Heslington, York, YO10 5DD, UK.*

[b] *York Neuroimaging Centre, The Biocentre, York Science Park, Innovation Way, Heslington, York, YO10 5DD, UK.*

1. **Experimental procedure**
2. **Catalyst details**
3. **Synthetic methods**
4. **NMR spectra example of the SABRE catalysts**
5. **Effect of polarisation transfer field on SABRE enhancements**
6. **Lifetime ($T_1$ and $T_{LLS}$) measurements**
7. **Parameters used to study theoretical model**
8. **Pulse sequence to achieve in-phase singlet derived signal.**
9. **References**

## 1. Experimental procedure

All NMR measurement were carried out on 400 MHz Bruker Avance III spectrometer. Depending on requirements, two different methods were used to study the SABRE process as described below.

**Shake & Drop:** Para-hydrogen was produced by cooling $H_2$ gas over a spin-exchange catalyst ($Fe_2O_3$) at 30K temperature. This method was able to provide para-hydrogen with more than 93% purity. Samples involved in the analysis were prepared with different batch of concentrations (listed below) dissolved in 0.6 ml deuterated Methanol solvent in a 5 mm NMR tube fitted with a J. Young's tap. The resulting solutions were then degassed by 3 cycles of freeze-pump-thaw method before filling the tube with *p*-$H_2$ at a pressure of 3 bar. Once filled with *p*-$H_2$, the tubes were shaken vigorously for ~10 seconds in a fringe field of ~65 Gauss around a 9.4 T Bruker spectrometer. Immediately after that, tubes were rapidly transported inside the spectrometer for subsequent NMR detections.

**Flow:** Magnetic field dependency studies of SABRE were done using more controlled 'Flow' method. The system consists of a mu-metal shielded chamber which stores samples at desired magnetic field and also connected to *p*-$H_2$ source to bubble inside it. Samples were prepared with same concentrations as were in the Shake & Drop method described earlier. Systematic studies were done by bubbling *p*-$H_2$ for 10 s at different magnetic mixing fields (from 0 G to 150 G) before transferring it in to the 9.4 T magnet for successive NMR observations.

**Enhancement factor:** This factor was calculated by taking the ratio of the integrals of peaks in the hyperpolarised spectra and thermal equilibrium spectra.

**Sample preparation:** A set of typical conditions used in the sample preparatory are listed below. These were used in 'shake & drop' type experiments.

*Sample A:* 3.12 μM (2 mg) of catalyst + 15.56 μM (1.93 mg) of **1** (or **2**) (thus making a 1:5 ratio) in 0.6 ml of solvent.

*Sample B:* 3.12 μM (2mg) of catalyst + 62.24 μM (7.72 mg) of **1** (or **2**) (thus making a 1:20 ratio) in 0.6 ml of solvent.

*Sample C:* 3.12 μM (2 mg) of catalyst + 15.56 μM (2.17 mg) of **3** (thus making a 1:5 ratio) in 0.6 ml of solvent.

*Sample D:* 3.12 μM (2 mg) of catalyst + 62.24 μM (8.68 mg) of **3** (thus making a 1:5 ratio) in 0.6 ml of solvent.

For the 'flow' type experiments, we used 3 ml of sample volumes whilst maintaining the same relative concentrations.

## 2. Catalyst details

In the studies detailed here we used [IrCl(COD)(IMes)] as the catalyst precursor which was synthesized in our laboratory according to a literature procedure[1] [IMes = 1,3-bis(2,4,6-trimethyl-phenyl)imidazole-2-ylidene and COD = *cis,cis*-1,5-cyclooctadiene]. This reaction is shown below.

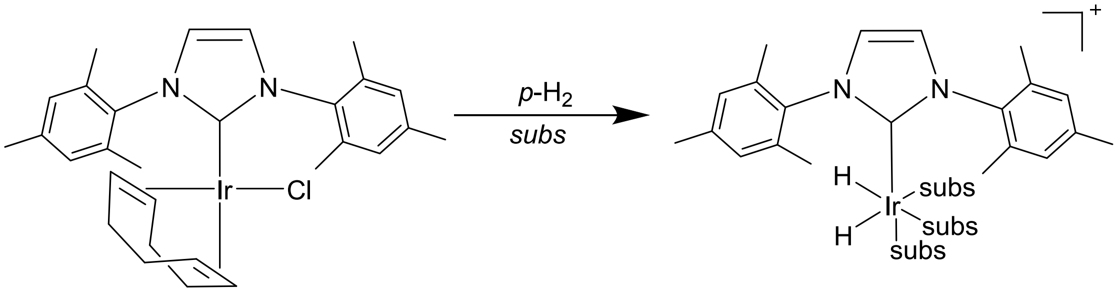


*Figure S1: SABRE reaction pathways. $Cl^-$ and Cyclooctadane omitted from the reaction product for clarity. 'subs' represents substrates **1**, **2** or **3**.*

## 3. Synthetic methods

### 3.1 General

Distilled water was employed where detailed. Brine refers to a saturated aqueous solution of NaCl. THF was freshly distilled from sodium and benzophenone ketyl or dried using a Grubbs solvent purification system. Petrol refers to the fraction of petroleum ether boiling in the range 40-60 °C. All reactions were carried out under $O_2$-free Ar or $N_2$ using oven-dried and/or flame-dried glassware.

Flash column chromatography was carried out using Fluka Chemie GmbH silica (220-440 mesh). Reverse-phase flash column chromatography was carried out using a Biotage Isolera with a SNAP-$C_{18}$-12g cartridge eluting with $H_2O$-MeCN containing 0.1% $NH_4OH$. Thin layer chromatography was carried out using Merck $F_{254}$ aluminium-backed silica plates. $^1H$ (400 MHz) and $^{13}C$ (100.6 MHz) NMR spectra were recorded on a Bruker-400 instrument with an internal deuterium lock. Chemical shifts are quoted as parts per million and referenced to $CHCl_3$ ($\delta_H$ 7.27), $(CH_3)_2SO$ ($\delta_H$ 2.54), $CDCl_3$ ($\delta_C$ 77.0) or $(CD_3)_2SO$ ($\delta_C$ 40.45). $^{13}C$ NMR spectra were recorded with broadband proton decoupling. $^{13}C$ NMR spectra were assigned using DEPT experiments. Coupling constants (*J*) are quoted in Hertz. Electrospray high and low resolution mass spectra were recorded on a Bruker Daltronics microOTOF spectrometer.

All commercial compounds listed were purchased from Sigma-Aldrich, Fluorochem or Alfa-Aesar and used as supplied unless otherwise stated. The following compounds were synthesised according to literature procedures; methyl-2,4-dichloronicotinate[2], ethyl-4,5,6-trichloronicotinate[3], and [IrCl(COD)(IMes)].[1]

**3.2 Synthetic Methods and Characterisation**

**Ethyl-2,4-*d*$_2$-nicotinate , S1**

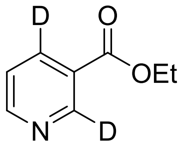


**S1**

5% Pd/C (14 mg, 10 wt%) was added to a stirred suspension of methyl-2,4-dichloronicotinate (135 mg, 0.65 mmol, 1.0 eq. and $K_2CO_3$ (250 mg, 2.75 mmol, 2.5 eq.) in EtOD (10 mL) in a 30 mL Parr Reactor. The Parr reactor was sealed, purged with $N_2$(g) and pressurised with $D_2$(g) to 8 bar. The reaction was stirred at rt for 3 h. Then, the pressure was released and the suspension was filtered through Celite® and washed with $CH_2Cl_2$ (3 x 20 mL). The filtrate was concentrated under reduced pressure to give the crude product. Purification by flash column chromatography with 1:1 petrol-EtOAc as eluent gave ethyl-2,4-*d*$_2$-nicotinate **S1** (62 mg, 62%) as a pale yellow oil, $R_F$ (1:1 petrol-EtOAc) 0.3; **$^1$H NMR** (400 MHz, $CDCl_3$) $\delta$ (ppm) 8.77-8.75 (m, 1H), 7.38-7.37 (m, 1H), 4.41 (qd, $J$ = 7.2, 3.3 Hz, 2H), 1.42-1.38 (m, 3H); **$^{13}$C NMR** (101 MHz, $CDCl_3$) $\delta$ (ppm) 165.2 (s), 153.3 (s), 150.5 (t, $J$ = 28.1 Hz), 136.7 (t, $J$ = 25.1 Hz), 126.1 (s), 123.1 (s), 61.4 (s), 14.2 (s); **MS** (ESI) *m/z* 154 [(M + H)$^+$, 100] 126 [32]; **HRMS** (ESI) *m/z* [M + H]$^+$ calculated for $C_8H_8D_2NO_2$ 154.0832, found 154.0831 (+1.4 ppm error).

**2,4-*d*$_2$-Nicotinamide , 1**

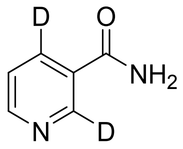


**1**

Ethyl 2,4-*d*$_2$-nicotinate **1** (62 mg, 0.406 mmol) was dissolved in ammonia (5 mL of a 7 N solution in MeOH) and the resulting solution was stirred at rt for 2 days. The solution was concentrated under reduced pressure to give the crude product. Purification by reverse phase flash column chromatography (0-30% MeCN) gave 2,4-*d*$_2$-nicotinamide **1** (50 mg, 100%) as a white solid, **$^1$H NMR** (400 MHz, *d*$_6$-DMSO) $\delta$ (ppm) 8.70 (CH, d, $J$ = 4.6 Hz, 1H), 8.18 (NH, br s, 1H), 7.62 (NH, br s, 1H), 7.50 (CH, d, $J$ = 4.6 Hz, 1H); **$^{13}$C NMR** (101 MHz, *d*$_6$-DMSO) $\delta$ (ppm) 166.9 (s), 152.4 (s), 148.8 (t, $J$ = 27.1 Hz), 135.3 (t, $J$ = 26.4 Hz), 129.9 (s), 123.8 (s); **MS** (ESI) *m/z* 147 [(M + Na)$^+$, 45] 125 [(M + H)$^+$, 100]; **HRMS** (ESI) *m/z* [M + H]$^+$ calculated for $C_6H_5D_2N_2O$ 125.0678, found 125.0681 (−1.8 ppm error).

**Methyl-2,6-*d*$_2$-nicotinate, S2**

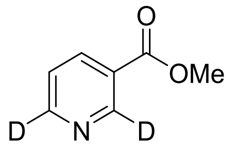

**S2**

5% Pd/C (10 mg, 10 wt%) was added to a stirred suspension of methyl-2,6-dichloronicotinate **S2** (100 mg, 0.485 mmol, 1.0 eq. and $K_2CO_3$ (167 mg, 1.21 mmol, 2.5 eq.) in THF (10 mL) in a 30 mL Parr Reactor. The Parr reactor was sealed, purged with $N_2$(g) and pressurised with $D_2$(g) to 8 bar. Then, the pressure was released and the suspension was filtered through Celite® and washed with $CH_2Cl_2$ (3 x 20 mL). The filtrate was concentrated under reduced pressure to give the crude product. Purification by flash column chromatography with 1:1 petrol-EtOAc as eluent gave methyl-2,6-$d_2$-nicotinate **S2** (43 mg, 63%) as a pale yellow solid, $R_F$ (1:1 petrol-EtOAc) 0.3; **$^1$H NMR** (400 MHz, $CDCl_3$) $\delta$ (ppm) 8.30 (CH, d, $J$ = 8.1 Hz, 1H), 7.40 (CH, d, $J$ = 8.1 Hz, 1H), 3.96 (s, 3H); **$^{13}$C NMR** (101 MHz, $CDCl_3$) $\delta$ (ppm) 165.8 (s), 153.1 (t, $J$ = 28.1 Hz), 150.5 (t, $J$ = 28.1 Hz), 137.1 (s), 125.9 (s), 123.2 (s), 52.4 (s); **MS** (ESI) $m/z$ 140 [(M + H)$^+$, 100]; **HRMS** (ESI) $m/z$ [M + H]$^+$ calculated for $C_7H_6D_2NO_2$ 140.0675, found 140.0674 (−1.8 ppm error).

**2,6-*d*$_2$-Nicotinamide, 2**

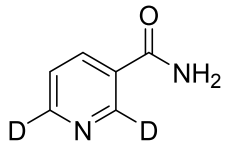

**2**

Methyl-2,6-$d_2$-nicotinate **2** (45 mg, 0.324 mmol) was dissolved in ammonia (6 mL of a 7 N solution in MeOH) and the resulting solution was stirred at rt for 2 days. Then, the solution was concentrated under reduced pressure to give the crude product. Purification by reverse phase flash column chromatography (0-30% MeCN) gave 2,6-$d_2$-nicotinamide **2** (34 mg, 85%) as a white solid, **$^1$H NMR** (400 MHz, $d_6$-DMSO) $\delta$ (ppm) 8.22 (CH, d, $J$ = 8.0 Hz, 1H), 8.21 (NH, br s, 1H), 7.62 (NH, br s, 1H) 7.50 (CH, d, $J$ = 8.0 Hz, 1H); **$^{13}$C NMR** (101 MHz, $d_6$-DMSO) $\delta$ (ppm) 166.9 (s), 152.0 (t, $J$ = 26.5 Hz), 148.8 (t, $J$ = 27.0 Hz), 135.7 (s), 130.0 (s), 123.8 (s); **MS** (ESI) $m/z$ 147 [(M + Na)$^+$, 72] 125 [(M + H)$^+$, 100]; **HRMS** (ESI) $m/z$ [M + H]$^+$ calculated for $C_6H_5D_2N_2O$ 125.0678, found 125.0679 (−0.7 ppm error).

**Methyl 3-chloropyrazine-2-carboxylate, S3**

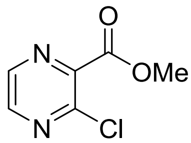

**S3**

(Trimethylsilyl)diazomethane (1.9 mL of a 2.0 M solution in diethyl ether, 3.78 mmol, 2.0 eq.) was added to a solution of 3-chloropyrazine-2-carboxylic acid (300 mg, 1.89 mmol, 1.0 eq.) in methanol (5 mL) and diethyl ether (5 mL). The reaction mixture was stirred at room temperature for 30 minutes and then concentrated under reduced pressure to give the crude product. Purification by flash column

chromatography with 1:1 petrol-EtOAc as eluent gave methyl 3-chloropyrazine-2-carboxylate **S3** (305 mg, 94%) as a light brown solid; **$^1$H NMR** (400 MHz, $CDCl_3$) $\delta$ (ppm) 8.38 (CH, d, $J$ = 2.4 Hz, 1H), 8.33 (CH, d, $J$ = 2.4 Hz, 1H), 3.78 (s, 3H); **$^{13}$C NMR** (101 MHz, $CDCl_3$) $\delta$ (ppm) 163.2 (s), 146.9 (s), 145.4 (s), 143.7 (s), 141.6 (s), 52.9 (s); **MS** (ESI) *m/z* 173 [$(M + H)^+$, 100], 175 [45], 141 [42], 143 [11]; **HRMS** (ESI) *m/z* $[M + Na]^+$ calculated for $C_6H_5ClN_2O_2$ 194.9932, found 194.9927 (+2.3 ppm error).

**Methyl 3-*d*-pyrazine-2-carboxylate, 3**

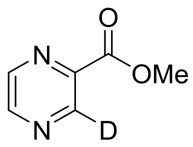


**3**

5% Pd/C (27 mg, 10 wt%) was added to a stirred suspension of methyl 3-chloropyrazine-2-carboxylate (270 mg, 1.56 mmol, 1.0 eq.) and $K_2CO_3$ (500 mg, 3.6 mmol, 2.3 eq.) in THF (8 mL) and $D_2O$ (2 mL) in a 30 mL Parr reactor. The reactor was sealed, purged with $N_2$, then pressurised with $D_2$ (8 bar). The reaction mixture was stirred at room temperature for 3 hours. The pressure was released and the suspension was filtered through Celite and washed with MeOH. The filtrate was concentrated under reduced pressure to give the crude product. Purification by flash column chromatography with 1:1 petrol-EtOAc as eluent gave methyl 3-*d*-pyrazine-2-carboxylate **3** (72 mg, 33%) as a colourless solid; **$^1$H NMR** (400 MHz, MeOD) $\delta$ (ppm) 8.86 (CH, d, $J$ = 2.4 Hz, 1H), 8.77 (CH, d, $J$ = 2.5 Hz, 1H), 4.04 (s, 3H); **$^{13}$C NMR** (101 MHz, $CDCl_3$) $\delta$ (ppm) 164.4 (s), 147.8 (s), 146.0 (t, $J$ = 28.7 Hz), 144.5 (s), 143.2 (s), 53.2 (s); **MS** (ESI) *m/z* 140 [$(M + H)^+$, 100], 108 [31]; **HRMS** (ESI) *m/z* $[M + Na]^+$ calculated for $C_6H_5DN_2O_2$ 162.0384, found162.0381 (+2.1 ppm error).

## 4. NMR spectra example of the SABRE catalysts

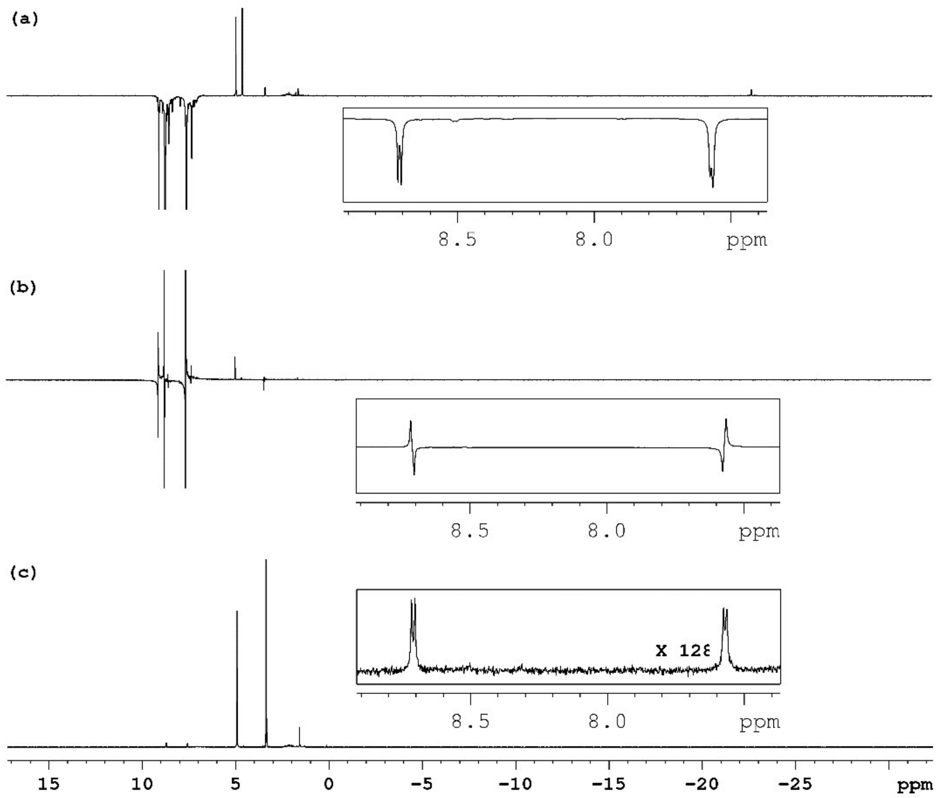


*Figure S2: (a) Full scale $^1$H NMR spectra of **1** after SABRE at 65 G; (b) SABRE-LLS detection after 1 s of spin-lock; (c) thermally realised NMR spectrum. Spectra include resonances from IMes, COD and hydride sites. Inset figures show magnified resonances for the two substrate spins in each cases.*

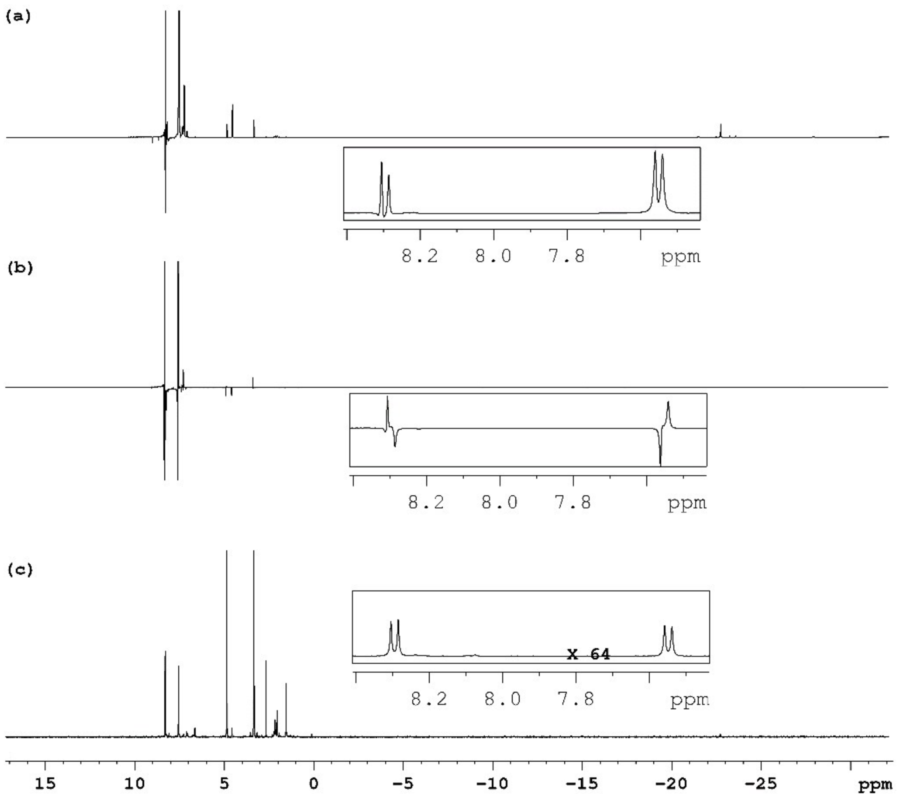


*Figure S3: (a) Full scale $^1$H NMR spectra of **2** after SABRE at 10 G; (b) SABRE-LLS detection after 1 s of spin-lock; (c) thermally realised NMR spectrum. Spectra include resonances from IMes, COD and hydride sites. Inset figures show magnified resonances for the two substrate spins in each cases.*

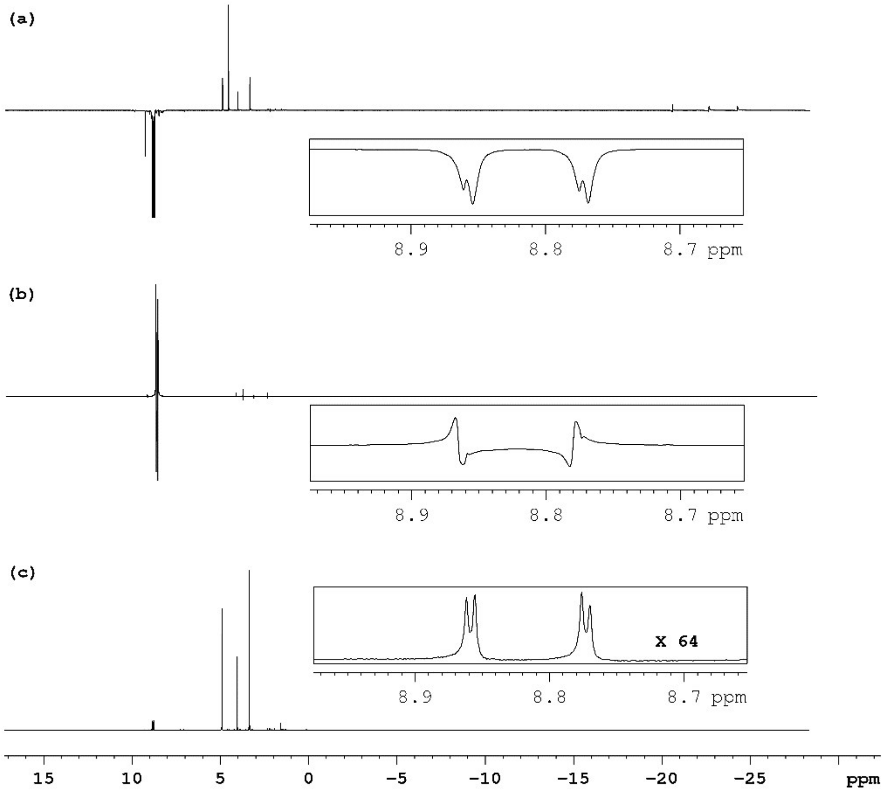


*Figure S4: (a) Full scale $^{1}H$ NMR spectra of **3** after SABRE at 65 G; (b) SABRE-LLS detection after 1 s of spin-lock; (c) thermally realised NMR spectrum. Spectra include resonances from IMes, COD and hydride sites. Inset figures show magnified resonances for the two substrate spins in each cases.*

The Fig. S2, S3 and S4 display identical measurements in the three substrates. Enhancement factors are reported in the next section.

## 5. Effect of polarisation transfer field on SABRE enhancements

Field dependence studies of the level of SABRE were completed in an automated ‘flow’ system, which permits repeated hyperpolarisation of samples at a constant and accurate field. The fields were varied from 0 to 150 G in increments of 10 G. The ‘flow’ system offers highly stable experimental condition both inside and outside of the NMR magnet and hence the results obtained are highly reproducible.

The corresponding values are detailed pictorially in Fig. S5, S6 and S7 for the three substrates in two different concentrations.

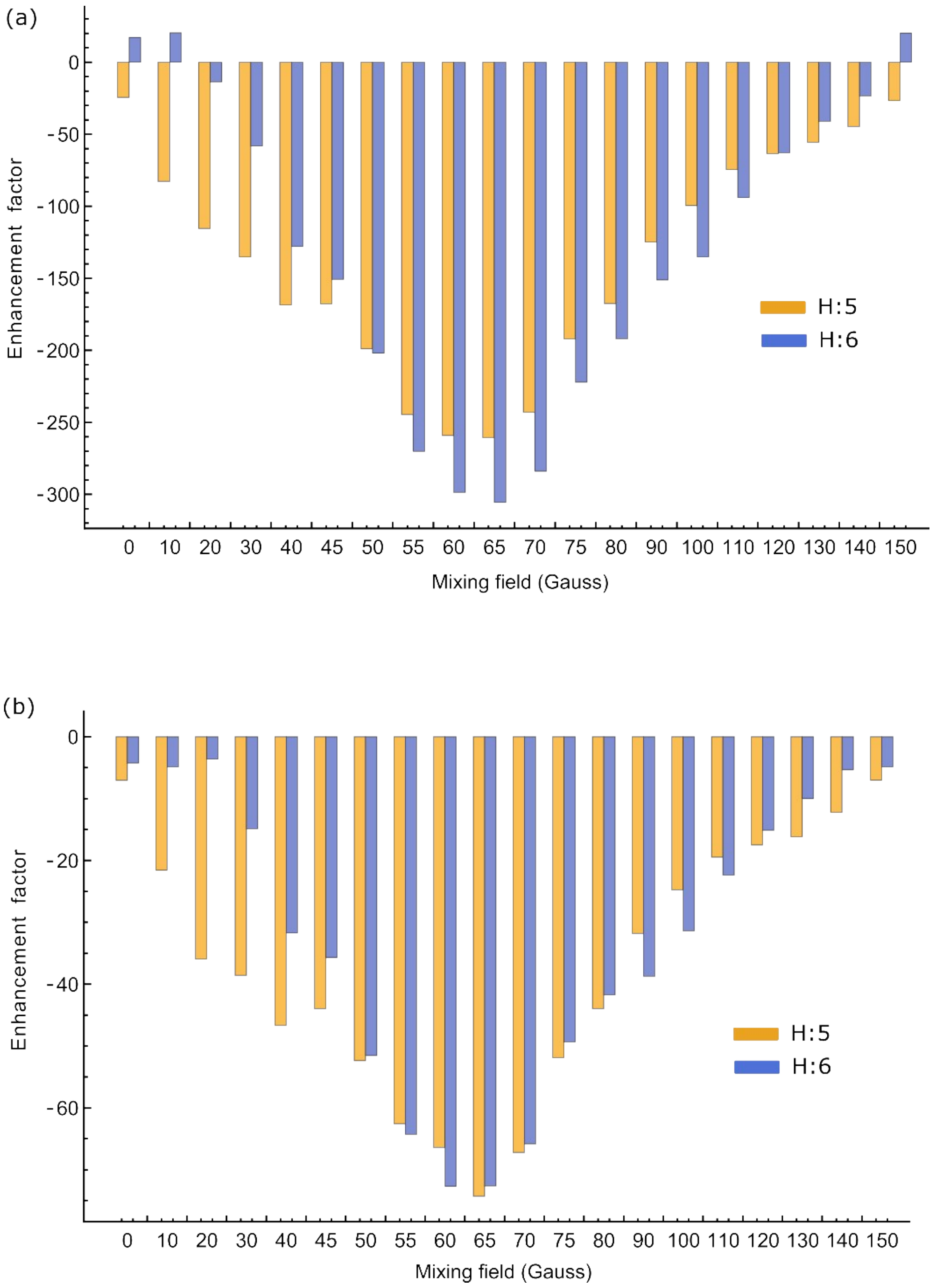


*Figure S5: Absolute $^{1}$H SABRE enhancements of substrate **1**, relative to thermal polarisation, for 5 equivalents (a) and 20 equivalents (b) of the substrate based on studies with the Ir(IMes)(COD)Cl catalyst.*

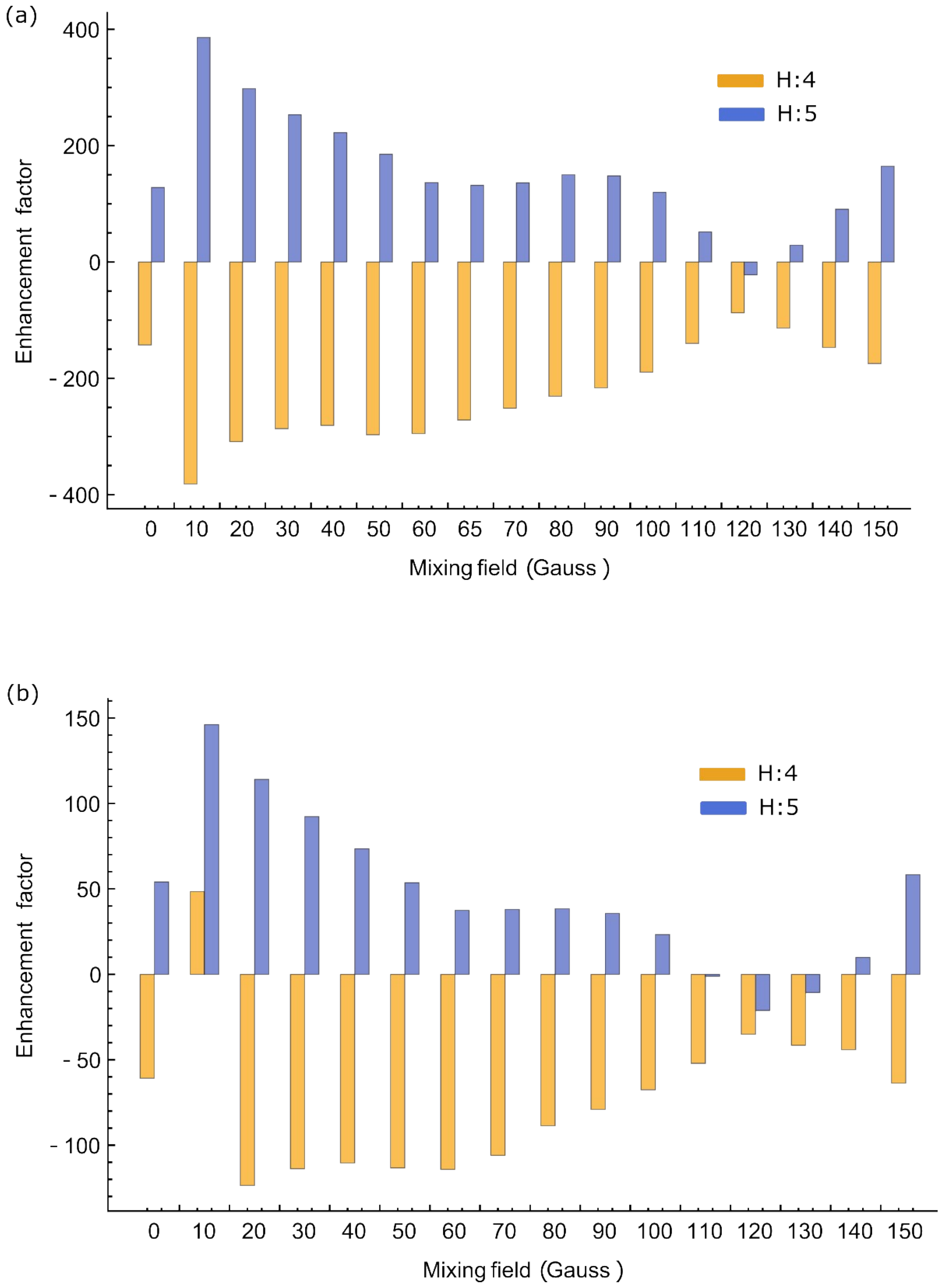


*Figure S6: Absolute [1]H SABRE enhancements of substrate **2**, relative to thermal polarisation, for 5 equivalents (a) and 20 equivalents (b) of the substrate based on studies with the Ir(IMes)(COD)Cl catalyst.*

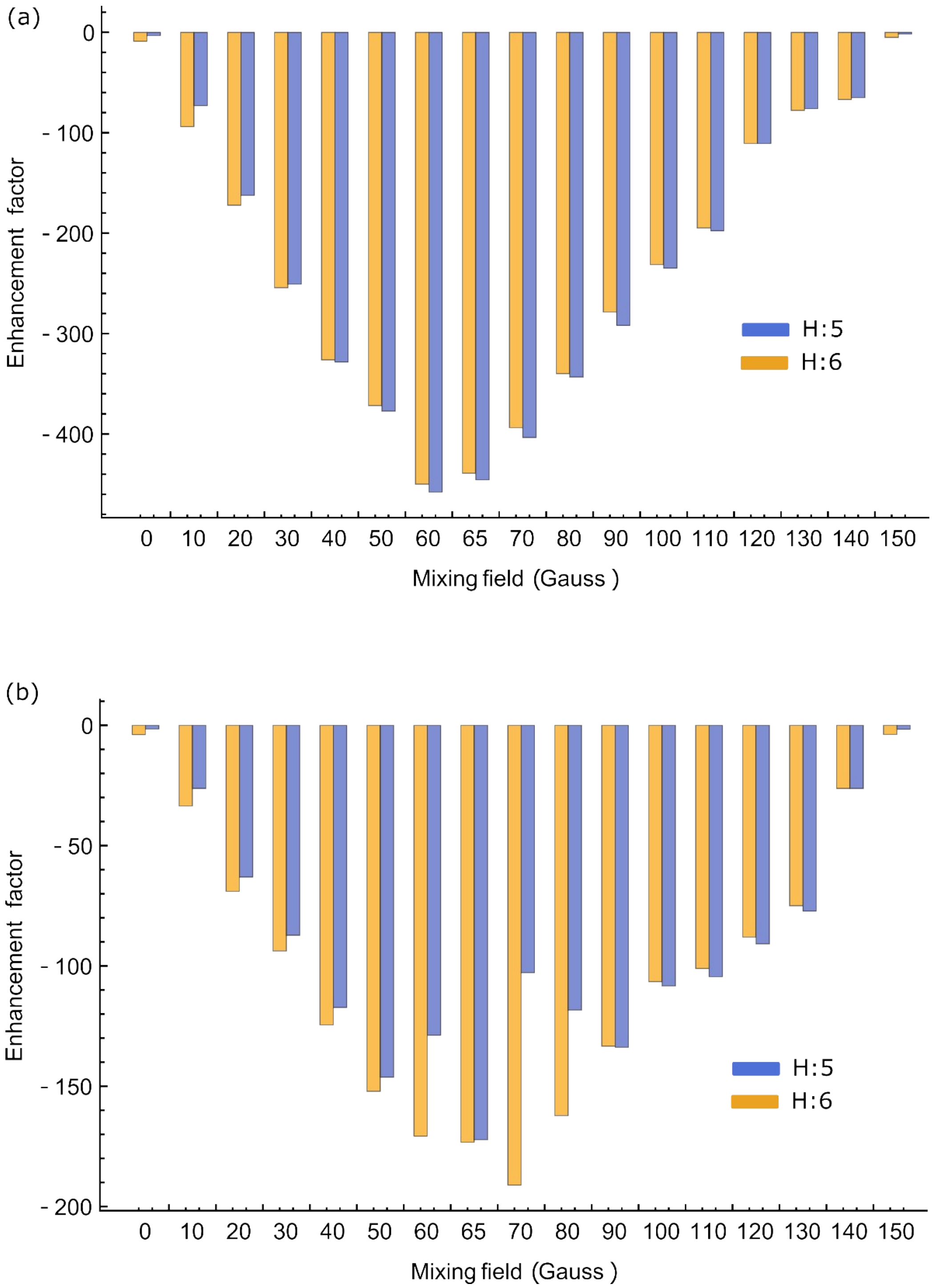


*Figure S7: Absolute $^{1}$H SABRE enhancements of substrate **3**, relative to thermal polarisation, for 5 equivalents (a) and 20 equivalents (b) of the substrate based on studies with the Ir(IMes)(COD)Cl catalyst.*

## 6. Lifetime ($T_1$ and $T_{LLS}$) measurements

$T_1$ measurements were completed using a standard inversion recovery experiment. Integrated data points are fitted to the standard equation: $M_z(\tau) = M_0(1 - 2e^{-\tau/T_1})$, where $M_z(\tau)$ are the integrated amplitudes at times $\tau$ and $M_0$ is a constant.

$T_{LLS}$ measurements are done by varying the singlet storage times (spin-lock durations) while keeping all other parameters unchanged during the experiment. Integrated amplitudes are fitted to a simple mono-exponential equation: $M_z(\tau) = M_0 e^{-\tau/T_{LLS}}$, where $M_z(\tau)$ are the integrated amplitudes at times τ and $M_0$ is a constant. In some cases the first points are dropped to improve the fit.

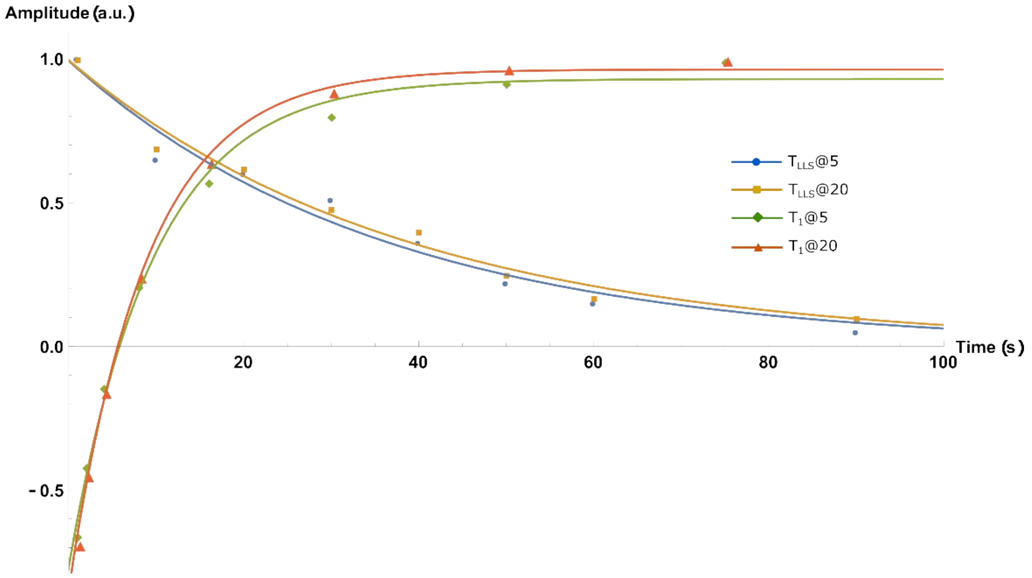


*Figure S8: Integrated relative signal amplitudes fitted to the previously detailed equations for substrate **1** based on samples with 5 and 20 equivalents of iridium. Inset caption details experimental type and sample specification. Fitted $T_1$ values (average of two spins) are 9.7 ± 1 s and 9.2 ± 0.5 s for the two cases. Fitted $T_{LLS}$ values are 37 ± 4 s and 38 ± 6 s for the two cases respectively.*

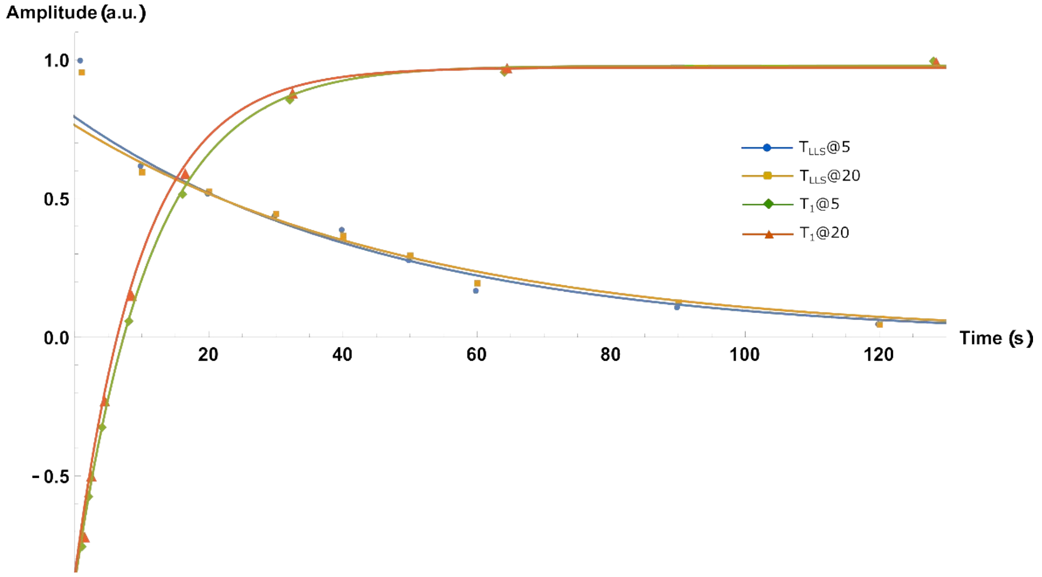


*Figure S9: Integrated relative signal amplitudes fitted to the previously detailed equations for substrate **2** based on samples with 5 and 20 equivalents of iridium. Inset caption details experimental type and sample specification. Fitted $T_1$ values (average of two spins) are 11.1 ± 0.3 s and 10.0 ± 0.4 s for the two cases. Fitted $T_{LLS}$ values are 47 ± 6 s and 49 ± 8 s for the two cases respectively.*

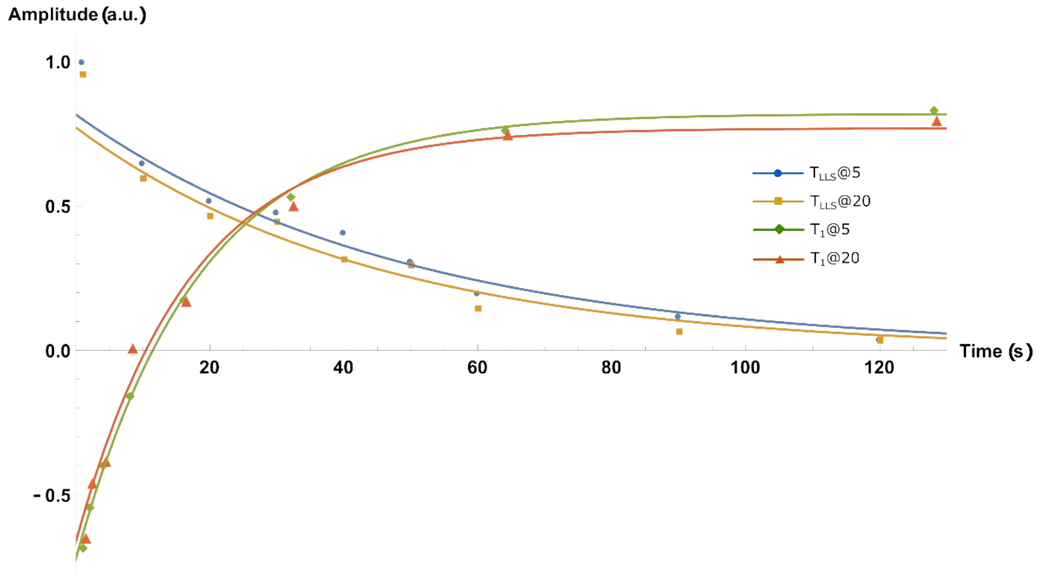


*Figure S10: Integrated relative signal amplitudes fitted to the previously detailed equations for substrate **3** based on samples with 5 and 20 equivalents of iridium. Inset caption details experimental type and sample specification. Fitted $T_1$ values (average of two spins) are 18.1 ± 1.1 s and 16.8 ± 2.6 s for the two cases. Fitted $T_{LLS}$ values are 49 ± 7 s and 45 ± 8 s for the two cases respectively.*

## 7. Parameters used to study theoretical model

*Table S1: Parameters used in the theoretical model described in the main text. The hydride spins are labelled as **I** and **S**, whereas substrate spins are labelled as **R** and **T**.*

| *Parameters* | | *Substrate* | | |
|---|---|---|---|---|
| | | **1** | **2** | **3** |
| *Chemical shifts (ppm)* | $\delta_I$ (ppm) | -22.70 | -22.50 | -20.60 |
| | $\delta_S$ (ppm) | -22.70 | -22.50 | -20.60 |
| | $\delta_R$ (ppm) | 7.57 | 7.60 | 8.77 |
| | $\delta_T$ (ppm) | 8.71 | 8.35 | 8.86 |
| *J-coupling constants (Hz)* | $J_{IS}$ (Hz) | 7.0 | 7.0 | 7.0 |
| | $J_{IR}$ (Hz) | 2.8 | 0.5 | 2.5 |
| | $J_{IT}$ (Hz) | 0.3 | 0.0 | 0.3 |
| | $J_{SR}$ (Hz) | 0.0 | 0.2 | 0.0 |
| | $J_{ST}$ (Hz) | 0.2 | 0.0 | 0.1 |
| | $J_{RT}$ (Hz) | 4.9 | 8.0 | 2.5 |

## 8. Pulse sequence to achieve in-phase singlet derived signal.

A modified version of LLS pulse sequence is used to detect in-phase singlet derived spectra.

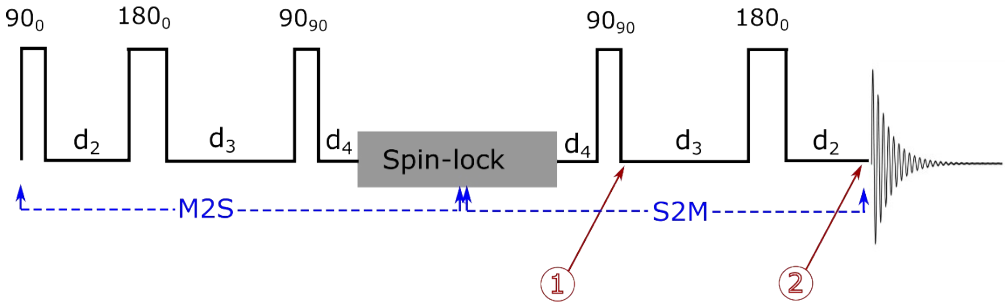


*Figure S11: Pulse sequence to achieve in-phase singlet derived signal. The standard LLS sequence detects anti-phase signal at time point 1. Extending the sequence allows in-phase signal detection at time point 2. Applied flip angle pulses are represented in standard convention. The first part of the sequence transfer the magnetisation into the singlet (M2S) and the middle section converts it back into magnetisation (M2S) for observation.*

Parameters used in the pulse sequence depend on individual spin systems with $d_2 = {}^1/_{4J}$, $d_3 = {}^1/_{4J} + {}^1/_{2\Delta\nu}$ and $d_4 = {}^1/_{4\Delta\nu}$, where J and Δv denote scaler-coupling constant and chemical shift difference between two spins in Hertz respectively. We used a Waltz-16 composite pulse of 1 KHz amplitude as the spin-lock in all cases.

The pulse sequence shown above works as follow:

Time point 1: Levitt's pulse sequence[4] converts the initial magnetisation into a singlet (M2S) and a brief readout sequence is used to acquire a typical anti-phase pattern for singlet derived magnetisation (see Fig. S6b).

$$\hat{R}_z + \hat{T}_z \xrightarrow{90_0 - d_2 - 180_0 - d_3 - 90_{90} - d_4} -2\hat{R}_x\hat{T}_x - 2\hat{R}_y\hat{T}_y \,(= |S_0\rangle + |T_0\rangle) \qquad \text{(S1)}$$

$$|S_0\rangle \xrightarrow{d_4 - 90_0} 2\hat{R}_x\hat{T}_z - 2\hat{R}_z\hat{T}_x - 2\hat{R}_y\hat{T}_y \qquad \text{(S2)}$$

Time point 2: Additional pulses convert the anti-phase magnetisation into in-phase signal (see Fig. S6c).

$$2\hat{R}_x\hat{T}_z - 2\hat{R}_z\hat{T}_x - 2\hat{R}_y\hat{T}_y \xrightarrow{d_3 - 180_0 - d_2} \hat{R}_y + \hat{T}_y + 2\hat{R}_y\hat{T}_y \qquad \text{(S3)}$$

Spindynamica[5] simulations confirm the efficiency of the pulse sequence to be high. Figure S12 shows experimental and simulated spectra, side-by-side in (a) a π/2 acquired spectra; and at (b) time point's 1 (c) and 2 respectively.

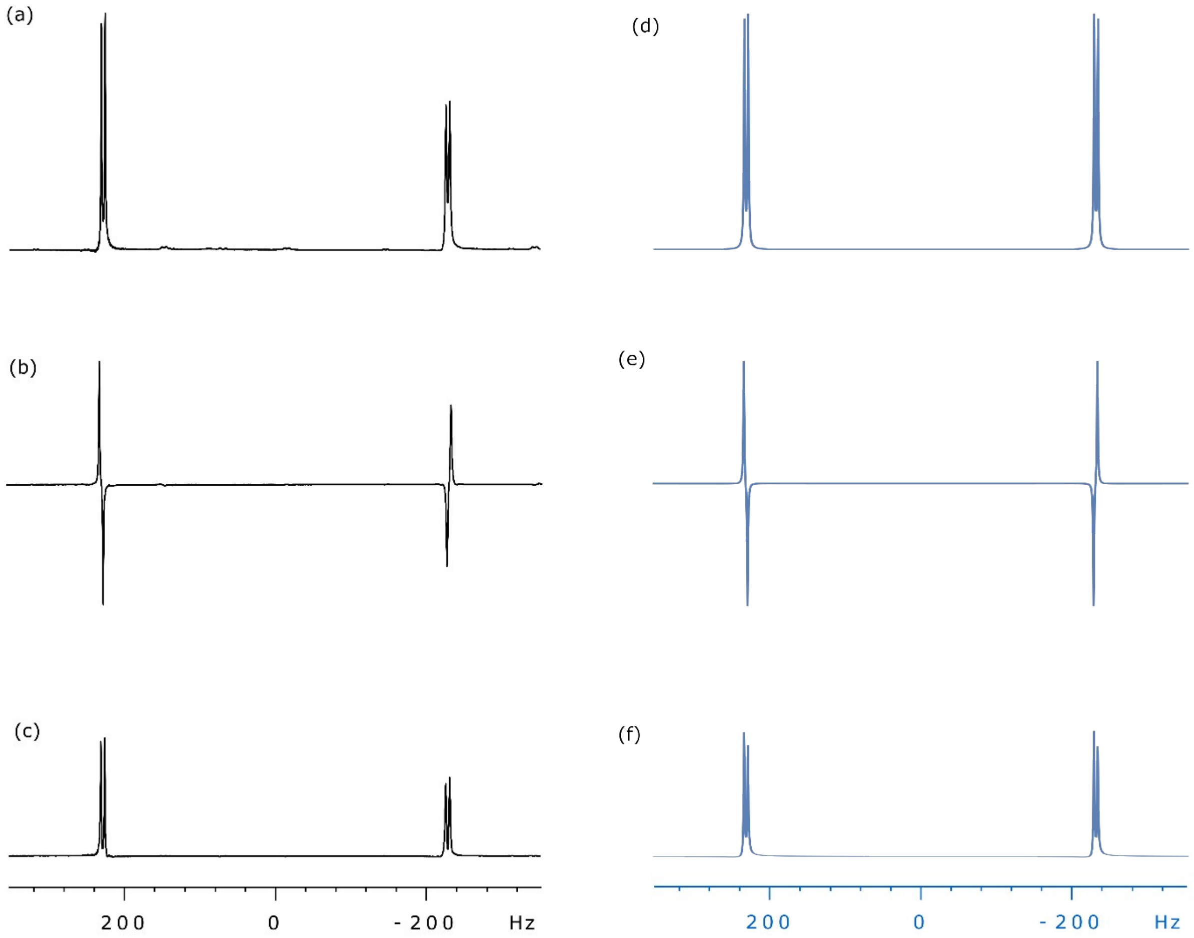


*Figure S12: $^{1}$H spectra of **1** at different stages of the experiment of Fig. S11. Spectra acquired on a 400 MHz spectrometer by (a) applying a π/2 pulse; (b) singlet derived anti-phase signal at time point 1; and finally (c) in-phase singlet derived signal is acquired at the end of the pulse sequence- time point 2. All spectra are shown on the same scale. Experimental spectra match the corresponding simulated spectra (d), (e), and (f) respectively.*

## 7. References


(1) Vazquez-Serrano, L. D.; Owens, B. T.; Buriak, J. M. *Inorganica Chimica Acta* **2006**, *359*, 2786.

(2) Khoje, A. D.; Gundersen, L.-L. *Tetrahedron Lett.* **2011**, *52*, 523.

(3) Wallace, E. M.; Lyssikatos, J.; Blake, J. F.; Seo, J.; Yang, H. W.; Yeh, T. C.; Perrier, M.; Jarski, H.; Marsh, V.; Poch, G.; Livingston, M. G.; Otten, J.; Hingorani, G.; Woessner, R.; Lee, P.; Winkler, J.; Koch, K. *J. Med. Chem.* **2005**, *49*, 441.

(4) Carravetta, M.; Levitt, M. H. *Journal of the American Chemical Society* **2004**, *126*, 6228.

(5) Levitt, M. H., SpinDynamica, <http://www.Spindynamica.soton.ac.uk>, 2016.
